# Role of FinTech and technological innovation towards energy, growth, and environment nexus in G20 economies

**DOI:** 10.1038/s41598-025-02794-2

**Published:** 2025-06-12

**Authors:** Harshita Jangid, Debi Prasad Bal, Navuluru Venkata Muralidhar Rao

**Affiliations:** Birla Institute of Technology and Science (BITS), Pilani Campus, Rajasthan, India

**Keywords:** FinTech, Technological innovation, Energy-growth-environment nexus, Panel VAR, G-20, Environmental sciences, Environmental social sciences

## Abstract

The current global consumption scenario is characterized as an energy-intensive economic development, indicating a rising mismatch in the harmonious relationship between individuals and the environment. The mismatch is caused by unsustainable consumption practices that do not take into account long-term ecological repercussions. To address this mismatch, it is necessary to turn toward sustainable energy use, greener technologies, and more responsible resource management, with the goal of balancing human economic progress with environmental care. Therefore, this study examines the influence of FinTech and technological innovations on the energy-growth-environment nexus in the context of G-20 economies for the time span of 2005- 2022. The study employs the panel vector autoregressive (PVAR) model in the generalized method of moment (GMM) approach to explore the interrelationship among the variables. From the findings, it was concluded that FinTech has a positive impact on the energy-growth-environment nexus. Similar to FinTech, technological innovation also has favourable influence on the energy-growth-environment nexus. Finally, there exist positive influence of energy on growth and environment, whereas rising carbon emissions exerts negative influence on growth and renewable energy consumption. From the policy standpoint, authorities can catalyse a more sustainable and inclusive future by encouraging collaboration among the fintech, technological advancement, energy, and environmental sectors.

## Introduction

As one of the most pressing ecological issues of this decade, the detrimental impact of high energy consumption and rising carbon emissions continues to escalate quickly, profoundly impacting the environmental conditions as well as economies and their functioning. According to the IPCC 2023, developing countries will require $127 billion annually by 2030 and $295 billion by 2050 to adapt effectively^1^. In response, nations have committed to international accords aimed at reducing fossil fuel reliance and fostering sustainable development. A key initiative, the Paris Climate Agreement 2015, underscores the essential role of financial support in achieving a zero-emissions future^2^.

The interplay between energy consumption, economic growth, and environmental sustainability has become a central research focus. While economic and technological advancements have boosted GDP, they have also intensified energy demands often at the cost of ecological health^3^. However, the rapid rise of Fintech has introduced transformative innovations. Encompassing digital finance, blockchain, and decentralized platforms, FinTech offers practical, sustainable solutions that support efficient energy use and environmental preservation^4,5^. In recent years, global economies have undergone transformative shifts driven by major advancements in the banking sector and broader technological innovation^6,7^. FinTech, the fusion of digital technology with financial services, has revolutionized the finance sector, disrupting traditional institutions through cost-efficient, customer-centric solutions^8^. Concurrently, innovations in artificial intelligence, information & communication technology, and clean energy technologies such as carbon capture, smart grids, and energy storage are accelerating the transition to sustainable energy systems^9,10^. Together, FinTech and technological innovation hold significant potential to reshape the energy-growth-environment nexus^8^.

The integration of FinTech and technological innovation presents both opportunities and challenges for the energy-environment nexus. FinTech facilitates decentralized energy systems, improving access to renewable energy, promoting GDP growth, and reducing emissions^11–15^. Additionally, green finance and innovation—enabled by FinTech—encourage corporate adoption of sustainable practices, such as renewable energy use and efficient waste management^6,16^. However, the environmental impact of high fossil fuel consumption in some digital platforms raises concerns about overall energy efficiency^17,18^. Technological advancements play a vital role in addressing these issues by enabling data-driven, transparent, and efficient energy allocation^4^. Furthermore, artificial intelligence also has substantial effect on adoption of renewable energy^9,19^.

This study examines the impact of FinTech and technological innovation on the energy-growth-environment nexus within the G20 context. Representing 85% of global economic output and 75% of trade, G20 countries are pivotal in shaping global finance, economic development, and environmental governance^20^. As leaders in technological innovation and policy implementation particularly through smart cities and green infrastructure in Europe, North America, and East Asia; G20 nations are at the forefront of aligning economic growth with sustainability goals^20^. Exploring the intersection of FinTech, energy systems, and environmental policy within these economies provides critical insights into sustainable global development.

This paper contributes to existing research in three key ways. First, while prior studies have explored the impacts of FinTech and technological innovation on energy and environmental systems, they often focus solely on developed or developing nations, overlooking the unique context of G20 countries. As global leaders in economic output, energy consumption, and innovation, the G20 presents a critical, yet underexplored, framework for analysing the energy-growth-environment nexus. Second, the study employs a single-equation panel VAR approach, using Impulse Response Analysis to capture dynamic interactions and assess the relative significance of each variable. This methodological framework enhances predictive accuracy and clarity in understanding variable interdependencies. Third, the paper offers actionable insights for policymakers, outlining strategic pathways for leveraging FinTech and technological advancement to drive sustainable economic growth, energy efficiency, and environmental protection. By examining this nexus through a G20 lens, the study highlights how digital finance and innovation can reshape sustainability efforts on a global scale.

The remainder of the paper content is arranged as follows: the literature review is covered in Section "Theoretical background and empirical literature", the data and methodology are displayed in Section "Data sources and methodology", the results analysis is covered in Section "Result and discussion", and the conclusion and policy implications are provided in Section "Conclusion and policy implication".

## Theoretical background and empirical literature

### Theoretical background

The role played by FinTech and technological innovation on the energy-environment-economic growth nexus is an evolving field of study that looks at how advances within financial services and technological developments influences economic growth, energy consumption, and environmental sustainability, and also the interrelationship among these variables. While energy consumption remains essential for economic development, it often leads to environmental degradation, including increased carbon emissions and resource depletion^21^. However, the rise of FinTech offers new opportunities to reconcile economic growth with environmental sustainability by enabling smarter, greener energy systems and more efficient resource use^4^.

In the context of economic growth, Everett Rogers’ (1995) innovation diffusion theory explains how new and innovative designs, as well as creative ideas for upgrading technologies, contribute to economic growth and advancements in financial products and services over time^22^. Endogenous Growth Theory supports the association between FinTech, technological innovation, and economic growth^23^. This theory explains how technological development, innovation, and human capital drive economic growth. FinTech and technical innovations render financial inclusion, literacy, and training more accessible to poor segments of society, leading to improved human capital^24,25^.

FinTech’s role in renewable energy is expanding alongside the global shift from fossil fuels to sustainable energy systems. Through innovations like P2P banking and crowdfunding, FinTech facilitates financing for green initiatives, enhances energy trading, and supports the integration of clean energy particularly in emerging economies such as China^18,26^. These developments contribute to both environmental sustainability and economic progress. However, the sector’s technological intensity, especially in cryptocurrency mining raises environmental concerns. Bitcoin mining, for instance, demands substantial energy due to blockchain operations, leading to significant carbon emissions and resource strain despite its financial innovation potential^12,17^.

**Fig. 1:**
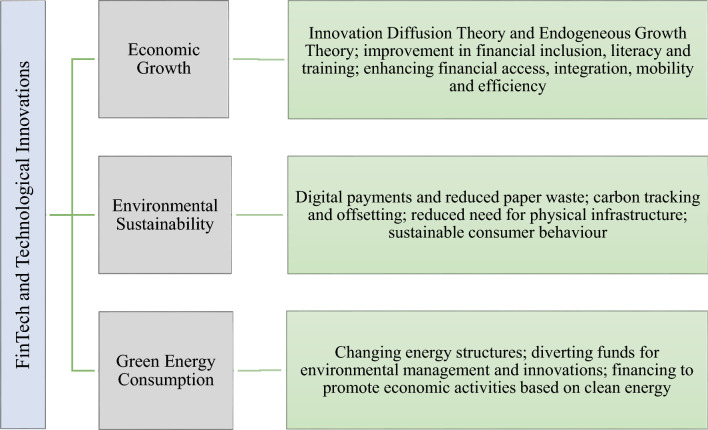
Graphical explanation depicting how FinTech and Technological Innovation impact economic growth, environmental sustainability and green energy consumption.

The graphical explanation depicting how FinTech and Technological Innovation impact economic growth, environmental sustainability and green energy consumption is shown in Fig. 1.

### Empirical literature

In this section, we focus on the empirical studies pertaining to the influence of FinTech and technological innovation on energy consumption, economic growth, and environmental sustainability.

#### FinTech and energy-economy-environment nexus

Existing literature on the nexus between FinTech, energy consumption, economic growth, and environmental sustainability can be broadly categorized into three streams: (1) the impact of FinTech on economic growth, (2) its role in reducing carbon emissions and improving environmental quality, and (3) its contribution to renewable energy adoption.

First, multiple studies affirm FinTech’s positive effect on economic growth^24,27–29^. Research shows that mobile and internet technologies promote economic development, particularly in low-income countries^30^. However, some findings reflect nonlinear relationships.^31^ note an inverted U-shaped link between financial development and productivity, while^32^ observe a U-shaped relationship between FinTech and growth. Similarly,^33^ found no significant connection in China using a VAR model. Others highlight FinTech’s role in poverty reduction, financial inclusion, and resilience during economic shocks like the COVID-19 pandemic^29,34–36^.

Second, FinTech has been recognized as a key driver in supporting green energy transitions and reducing carbon emissions. By enabling financing for clean energy projects and enhancing environmental protection, FinTech helps mitigate climate-related financial risks^12,37–41^ emphasize that unchecked emissions can ultimately hinder economic growth, further reinforcing the importance of FinTech in climate strategies. However, some studies caution that FinTech may also contribute to ecological harm especially through energy-intensive processes like cryptocurrency mining^11,42,43^.

Lastly, the adoption of FinTech is increasingly linked to the promotion of renewable energy. It aids in financing transitions to cleaner sources and encourages long-term growth by fostering sustainable practices^5^. Yet, the environmental impact remains mixed due to technological energy demands, indicating an ambiguous overall effect that depends on implementation context and technological maturity.

From the above literature, we formulate our first hypothesis as:

##### Hypothesis 1

FinTech has positive impact on renewable energy consumption, economic growth and environmental sustainability.

#### Technological innovation and energy-economy-environment nexus

The relationship between technological innovation, energy consumption, economic growth, and environmental sustainability has largely been studied in isolation, typically through two main lenses: technological innovation and growth, and technological innovation and pollution.

On economic growth, prior literature overwhelmingly supports a positive relationship with technological innovation^44,45^. Innovation enhances productivity, improves living standards, and drives long-term economic development^46,47^ . Country-specific studies, such as^48^ for India and^49^ for Indonesia, affirm the significant role of green innovation and reindustrialization strategies in supporting sustainable growth. Likewise,^50^ found that digital technologies and financial inclusion enhance output across 84 countries.

Technological innovation also plays a pivotal role in environmental sustainability and green energy transitions. It enables efficient resource use in developing countries^51^ and reduces environmental degradation while fostering economic gains^52^. Studies highlight its positive effects on renewable energy adoption and carbon emission reduction^53,54^. For example,^5^ show that digital technologies in China improve energy efficiency and stimulate green tech innovation, while^55^ underscore the role of CCUS in combating climate change.

Hence, by analysis the above literature, we formulate our second hypothesis as:

##### Hypothesis 2

Technological Innovation has a positive impact on renewable energy consumption, economic growth, and environmental sustainability.

## Data sources and methodology

In this section, we will state the variables used in our study, their sources and measurements, and analytical framework as well as methodology employed for the analysis of the relationship among the variables.

### Data sources

The study consists of a panel data and adopts a secondary data sample covering annual data of G-20 nations, excluding the European Union due to data unavailability. Our study was conducted for the time-span of 2005 to 2022. The study period and sample size are determined considering data constraints for other periods and countries, and the data for automated teller machine was not available before 2005. This analysis incorporates data on the FinTech index comprising of ATMs, internet usage, mobile cellular subscription and fixed broadband subscription; environment sustainability is proxied by carbon emissions (CO_2_) as has been used by previous studies, such as,^56^; energy consumption is proxied by variable renewable energy consumption; and economic growth for each of the G-20 countries. We have gathered the data from the World Bank’s World Development Indicator (WDI) database. Table 1 shows description of the variables, their notion, and their sources.

**Table 1:** Description of the Variables. Source: Author’s own compilation.

Variables	Symbols	Measurement	Source
1. Carbon Emissions	CO_2_	CO_2_ emissions (metric tons per capita)	WDI
2. Economic Growth	GDPPC	GDP Per Capita (constant 2015 US$)	WDI
3. Energy Transition	REC	Renewable energy consumption (% of total final energy consumption)	WDI
4. FinTech Index	FinTech	Index construction using: Automated Teller Machines for every 100,000 adults Fixed Broadband Subscription per 100 individuals; Mobile Cellular Subscription per 100 individuals Individuals using the Internet (% of the population)	Author’s Construction; WDI
5. Technological Innovation	TI	Patent applications, residents	WDI

Note 1: WDI- World Development Indicators (https://databank.worldbank.org/source/world-development-indicators).

We changed all of our data to logarithmic form for two reasons. First and foremost, it can help remove any seasonality that may be present in the dataset. Second, each variable is in growth mode and can be compared to one another.

**Fig. 2:**
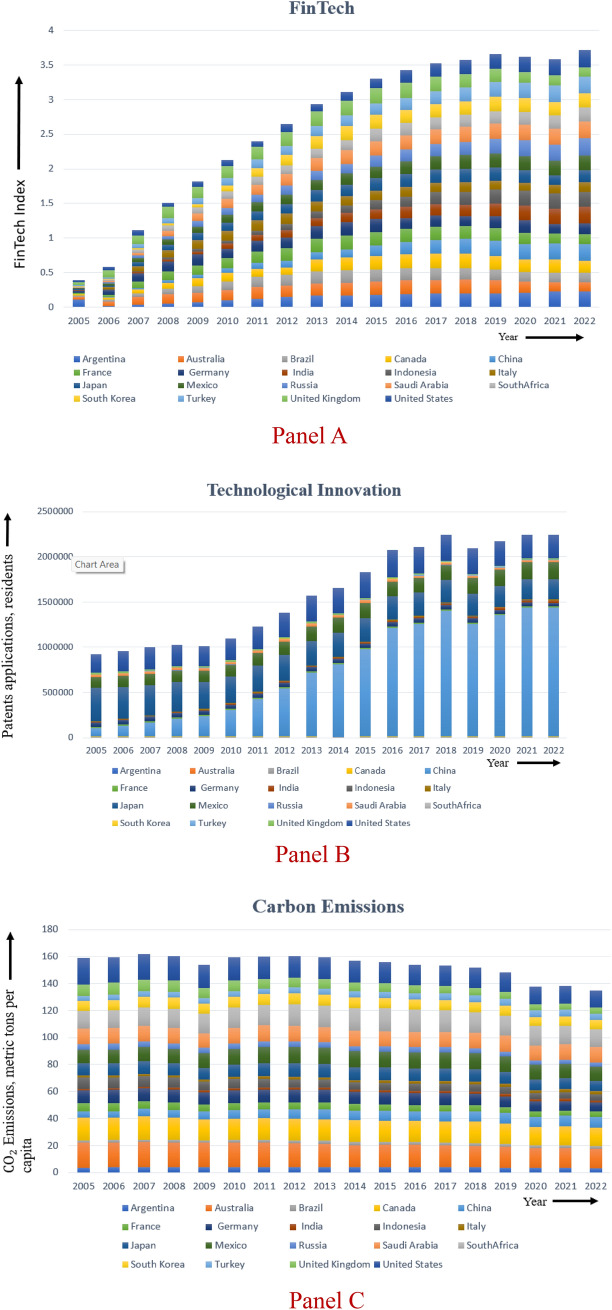
Graphs showing trends in G-20 economies for FinTech, technological innovation and carbon emissions.

Additionally, Fig. 2: panel A, panel B, and panel C has also been displayed to incorporate panel graphs showcasing FinTech, technological innovation, and carbon emissions, respectively. As observed from Fig. 2, there is an upward trend for FinTech (as constructed by authors using the variables for FinTech as given in Table 1), and technological innovations in G-20 economies, indicating a significant rise in FinTech services and technological advancements over the period of 2005 to 2022, whereas, a declining trend can be observed in the case of carbon emissions, indicating reduced carbon emissions over the period of time.

### Measurement of variables

#### Construction of FinTech index

For the construction of the FinTech index, our study relies on the Geometric mean method. This method of estimation was put together by Mahbub ul Haq in the 1990s to derive the country’s Human Development Index (HDI). The HDI values were calculated following two steps. Initially, the dimensional indices are generated, and then they are taken together.

In a similar vein, our study converted the variables into indices after first normalizing them. To do this, the indicators stated in various units are converted into indices between 0 and 1 by setting minimum and maximum values for each country and variable. The dimension indices are computed as follows when the lowest and maximum values have been established:

$$Dimension index= \frac{{X}_{A}- {X}_{MIN}}{{X}_{MAX}- {X}_{MIN}}$$

where X_A_, X_MIN_ and X_MAX_ represents actual value, minimum value and maximum value of the variables, respectively.

After all the variables have been transformed into sub-major indices with a scale of 0 to 1, we give them weights using an equal-weighted approach, meaning that each variable is given a weight of one-quarter. The FinTech index was then created by taking the geometric mean of the four indices.

$$FinTech Index= \sqrt[4]{ATM*MCS*FBS*Internet}$$

where the highest performance is denoted by a 1 and the lowest performance by a 0. An increase in the value of the FinTech index indeed represents an improvement in FinTech’s performance for G-20 Nations.

### Analytical framework

To investigate the relationships between the variables, our study makes use of the standard production function. The following is the production function:

1 $${\text{Y}} = f\left( {{\text{K}},{\text{L}}} \right)$$

Here, output, capital and labour are represented by Y, K and L respectively.

The equations are as follows;

2 $${\text{CO}}_{{2}} = \, f \, \left( {{\text{FinTech}},{\text{ TI}},{\text{ REC}},{\text{ GDPPC}}} \right)$$

Here, Cobb–Douglas production function is used to study the above equation for G-20 nations for period ranging from 2005 to 2022.

3 $${{CO}_{2}}_{t-1}={\alpha }_{t}{{CO}_{2}}_{t-1}^{\beta 1}{FinTech}_{t-1}^{{\beta }_{2}}{TI}_{t-1}^{{\beta }_{3}}{REC}_{t-1}^{\beta 4}{GDPPC}_{t-1}^{{\beta }_{5}}$$

where $\beta 1$, ${\beta }_{2}$, ${\beta }_{3}$,$\beta 4$ and ${\beta }_{5}$ represents the elasticity coefficients of the dependent variable of lagged period, FinTech, technological innovation, renewable energy consumption, and economic growth, respectively. Next, natural logarithms is taken on both sides of the equation. Logarithmic conversion of variables helps with seasonal adjustment, their magnitude changes and result interpretation with the use of elasticity.

#### Framework for panel VAR model

To investigate the relationships between the variables, we employed the panel vector autoregressive (PVAR) model in the generalized method of moment (GMM) approach. We used this approach due to its following advantages: First, this approach helps anticipate the shocks and behaviours of one variable on other variables for the 10-period horizon, as well as to demonstrate the patterns that exist among the variables. Second, this methodology allows for modelling interdependencies and feedback effects among multiple variables over time. Each variable in the system can be influenced by its own lagged values and the lagged values of other variables. Furthermore, this method is useful for addressing endogeneity problems, which arises due to the correlation between independent variables and the error term. This endogeneity issue gives inconsistent and biased estimates that might impact our model, and also accounts for unobserved individual heterogeneity^57^. The problem of heterogeneity can be understood as a situation wherein coefficients of the model vary systematically, and are not constant across individual or group in the panel, leading to biasedness in the estimation.

Following^58^, panel VAR equation is as follows:

4 $${Z}_{it}= {\alpha }_{0}+ \sum_{j=1}^{p}{\alpha }_{j} {Z}_{i,t-j}+ {\mu }_{i}+ {e}_{i,t}$$

$$i\in \left\{\text{1,2},3\dots , N\right\}, t\in \{\text{1,2},3\dots , {T}_{i }\}$$

where *z*_it_ depicts the set of dependent variables. $${\alpha }_{j}$$ depicts coefficient of vector to be estimated. μ_it_ and e_it_ represents the fixed effect error term and idiosyncratic error term, respectively. The model assumes that the summation of the fixed effect error term is zero and the E[e_it_^T^e_it_] = ∑. and E ($${e}_{it}^{T}{e}_{ij})$$ = 0, show the stability of the VAR model. It is stable when the ∝ ¯ matrix is strictly less than 1. After satisfying the stability condition, we move on to impulse response functions (IRFs) which helps with analysing the variable for future horizons. IRFs highlight the interdependence of all variables, provide insight into how shocks propagate across a system, and investigate if modifications to one variable have a cascade effect on other variables.

## Result and discussion

**Fig. 3:**
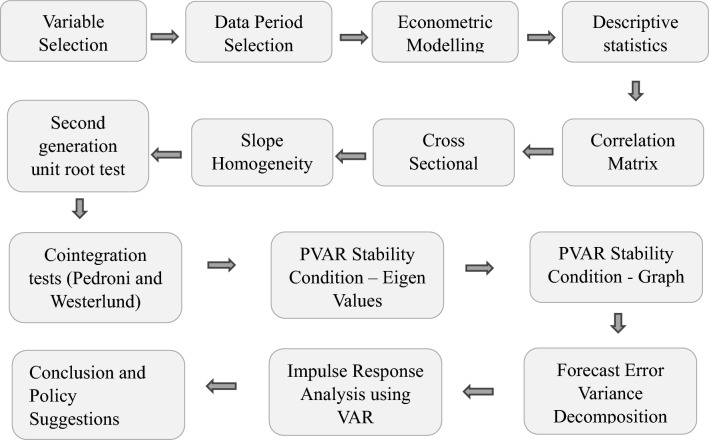
Flowchart showing Analytical Methodology.

In this section, we will focus on the preliminary analysis, result interpretation and discussion. The step-by-step procedure of the analysis and methodology used for the result findings is presented below (as shown in Fig. 3):

The summary statistics for our balanced panel data, providing an overview of the central tendency, spread, shape, and statistical significance of five variables are shown in Table 2. Our study contains a total of 342 observations with mean of CO_2_, FinTech, TI, REC and GDPPC as 5.83, 0.13, 3.98, 0.93, and 4.20 respectively. The spread or dispersion of the variable CO_2_, REC and GDPPC shows moderate variability, whereas FinTech and TI shows low and high spread around the mean, respectively. REC shows the most skewed distribution, with a high negative skew, and CO_2_ has a significant positive skew. The variables have varying degrees of normality, with CO_2_, FinTech, TI, REC, and GDPPC showing significant deviations from normality (based on the Jarque–Bera test). The results of the Jarque–Bera statistics thus show the rejection of the null hypothesis, which suggests a normal distribution. This finding highlights the necessity of a more potent approach to handling the non-normal distribution. As a result, our study deploys the GMM-PVAR strategy to deal with the non-normality situation. Next, the results obtained from the correlation matrix (as shown in Table 3) depicts the negative impact of FinTech and GDPPC on CO_2_ whereas this effect is positive in case of TI and REC.

**Table 2:** Descriptive Statistics. Source: Author’s own compilation.

	CO_2_	FinTech	TI	REC	GDPPC
Mean	5.831854	0.137566	3.989555	0.932856	4.209296
Median	5.684228	0.148788	3.934927	1.012837	4.291444
Maximum	7.058975	0.250000	6.154316	1.698970	4.804281
Minimum	5.164339	0.000000	2.075547	-1.000000	2.976700
Std. Dev	0.424644	0.064729	0.950877	0.571737	0.443357
Skewness	1.300592	-0.531597	0.314085	-1.926925	-0.695330
Kurtosis	4.071355	2.273823	2.272094	7.399514	2.713882
Jarque–Bera	112.7739	23.62246	13.17335	487.4623	28.72515
Probability	0.000000	0.000007	0.001379	0.000000	0.000001
Observations	342	342	342	342	342

**Table 3:** Correlation Matrix. Source: Author’s own compilation.

	CO_2_	FinTech	TI	REC	GDPPC
CO_2_	1.0000				
FinTech	-0.0179	1.0000			
TI	0.7399	0.1139	1.0000		
REC	0.0384	-0.0004	0.0641	1.0000	
GDPPC	-0.0943	0.2204	0.3056	-0.2791	1.0000

Next, our study employs cross-sectional dependency (CSD) test and the slope heterogeneity test in order to offer the basic framework for our analysis. Results for CSD test and slope heterogeneity test are given in Table 4. Results obtained points to the presence of cross-sectional dependency and slope heterogeneity in our analysis. Therefore, it is essential to look at the second-generation tests in the econometric assessment’s later portions in order to get over these challenges. We likewise opt for the second-generation CIPS unit root test. According to the CIPS test findings (as shown in Table 5), all indicators are stationary at the first difference at the 1% level of significance.

**Table 4:** Cross-Sectional Dependence Tests and Slope homogeneity. Source: Author’s own compilation.

Cross Sectional Dependence		Slope homogeneity	
Variables	CD-test (p-value)	Stat	Delta (p-value)
CO_2_	8.391 (0.000)	$\sim \Delta$	13.691 (0.000)
FinTech	45.501 (0.000)	$\sim \Delta$ adjusted	16.768 (0.000)
TI	12.653 (0.000)		
REC	10.239 (0.000)		
GDPPC	33.07 (0.000)

**Table 5:** Second generation unit root tests (CIPS). Source: Author’s own compilation.

	Level	1st Difference
CO_2_	-2.197	-3.505***
FinTech	-1.970	-3.782***
TI	-1.654	-4.126***
REC	-1.552	-3.177***
GDPPC	-2.033	-2.632***

We next verified whether or not there is cointegration among the variables before proceeding with result estimate. According to the Pedroni and Westerlund panel cointegration test results (as presented in Table 6), cointegration exists among the variables since all statistics reject the null hypothesis that there is no cointegration at the 1% and 5% significance levels.

**Table 6:** Cointegration Tests (Pedroni and Westerlund test). Source: Author’s own compilation.

Pedroni test for cointegration		Westerlund test for cointegration	
	Statistic (p-value)		Statistic (p-value)
Modified Phillips-Perron t	3.7455 (0.0001)	Variance ratio	-1.6797 (0.0465)
Phillips-Perron t	-1.3061 (0.0958)		
Augmented Dickey-Fuller t	-2.6092 (0.0045)		

We used the panel VAR approach for our results in the subsequent stage of our investigation.

**Fig. 4:**
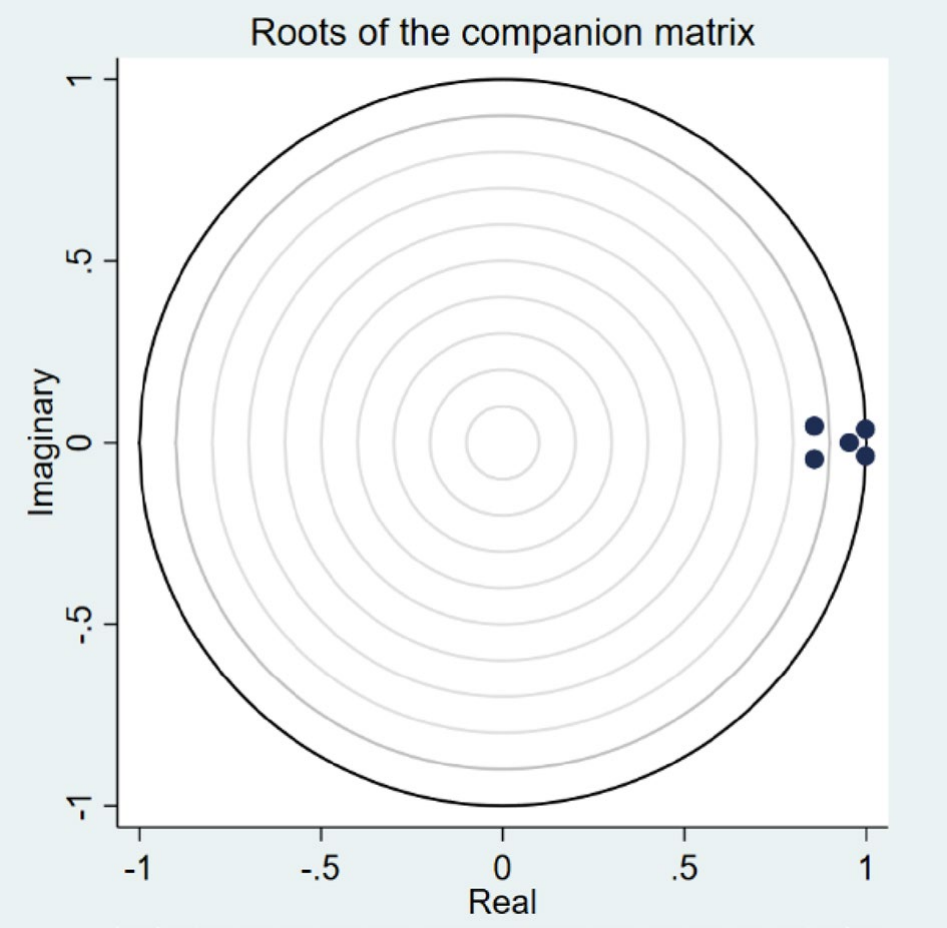
VAR Stability Graph.

First, as indicated in Table 7, we give the eigenvalue stability requirement for our models. This led us to the conclusion that the calculated eigenvalues satisfy our stability criterion as their values are less than 1. Additionally, Fig. 4 displays the VAR stability graph for our model. The following figure demonstrates that all of these eigenvalues fall inside the unit circle, proving the stationarity of our estimations and allowing us to proceed with the panel VAR model forecasting of the variance decomposition and impulse response function.

**Table 7:** Eigenvalue stability condition. Source: Author’s own compilation.

Eigen Values		
Real	Imaginary	Modulus
.9978066	-.037231	.998501
.9978066	.037231	.998501
.9530932	0	.9530932
.8568516	-.0455467	.8580613
. .8568516	.0455467	.8580613

All the eigenvalues lie inside the unit circle.

pVAR satisfies stability condition.

Next, our study uses Forecast error variance decomposition (FEVD) to demonstrate the inter-relationship among the variables. To find out how sensitive one indicator is to changes in another indication in the particular model over a long period of time, FEVD is used. Here, we showed the outcomes over the second, fifth, and tenth time-periods (as shown in Table 8). From the results obtained, it was observed that the forecast error variance of each variable is mostly impacted by its own historical shocks. Throughout all times, CO_2_ is heavily impacted by its own prior shocks, but after 5th period horizons, FinTech begins to have a major impact. The impact of FinTech on CO_2_ increased from 0.0136 over 5th horizon to 0.025 over 10th horizon period. Although CO_2_ has a significant influence, particularly in the immediate term (two periods ahead), FinTech is primarily self-explanatory. Similar to FinTech, TI is mostly described by its own shocks, with the other factors having little impact. With a small contribution from FinTech and TI, CO_2_ has a major impact on REC. The impact of FinTech on REC increases over the time with 0.0058 over 2nd horizon to 0.102 over 10th horizon. With CO_2_ having a significant impact at shorter timeframes, GDPPC exhibits a strong dependency on its own shocks. In addition, the impact of FinTech and TI on GDPPC shows increasing trend from 2nd horizon to 10th horizon.

**Table 8:** Forecast error variance decomposition (FEVD). Source: Author’s own compilation.

Response Variable		Impulse Variable				
	Periods-ahead	CO_2_	FinTech	TI	REC	GDPPC
CO_2_	2	.9902393	.0021498	.0060945	.0006445	.0008721
	5	.9134579	.0136803	.0630647	.0036199	.0061772
	10	.7424585	.0250356	.2116982	.0058019	.0150057
FinTech	2	.090811	.8896067	.0140688	.0032702	.0022433
	5	.2487124	.6131487	.1024078	.0219281	.0138029
	10	.4134748	.3491678	.1763558	.0392368	.0217649
TI	2	.0064905	.0010033	.9922689	.0000287	.0002087
	5	.004496	.0005127	.9926235	.0002435	.0021243
	10	.0037342	.0012056	.9869096	.0005818	.0075688
REC	2	.2158249	.0058217	.0140312	.7638268	.0004953
	5	.2058273	.0034916	.0201797	.7647315	.00577
	10	.1996563	.0102925	.016372	.7515472	.022132
GDPPC	2	.3068754	.0014066	.001668	.0012156	.6888344
	5	.2146411	.0080039	.0368593	.0130415	.7274543
	10	.1630445	.0253284	.2412425	.0520635	.518321

**Fig. 5:**
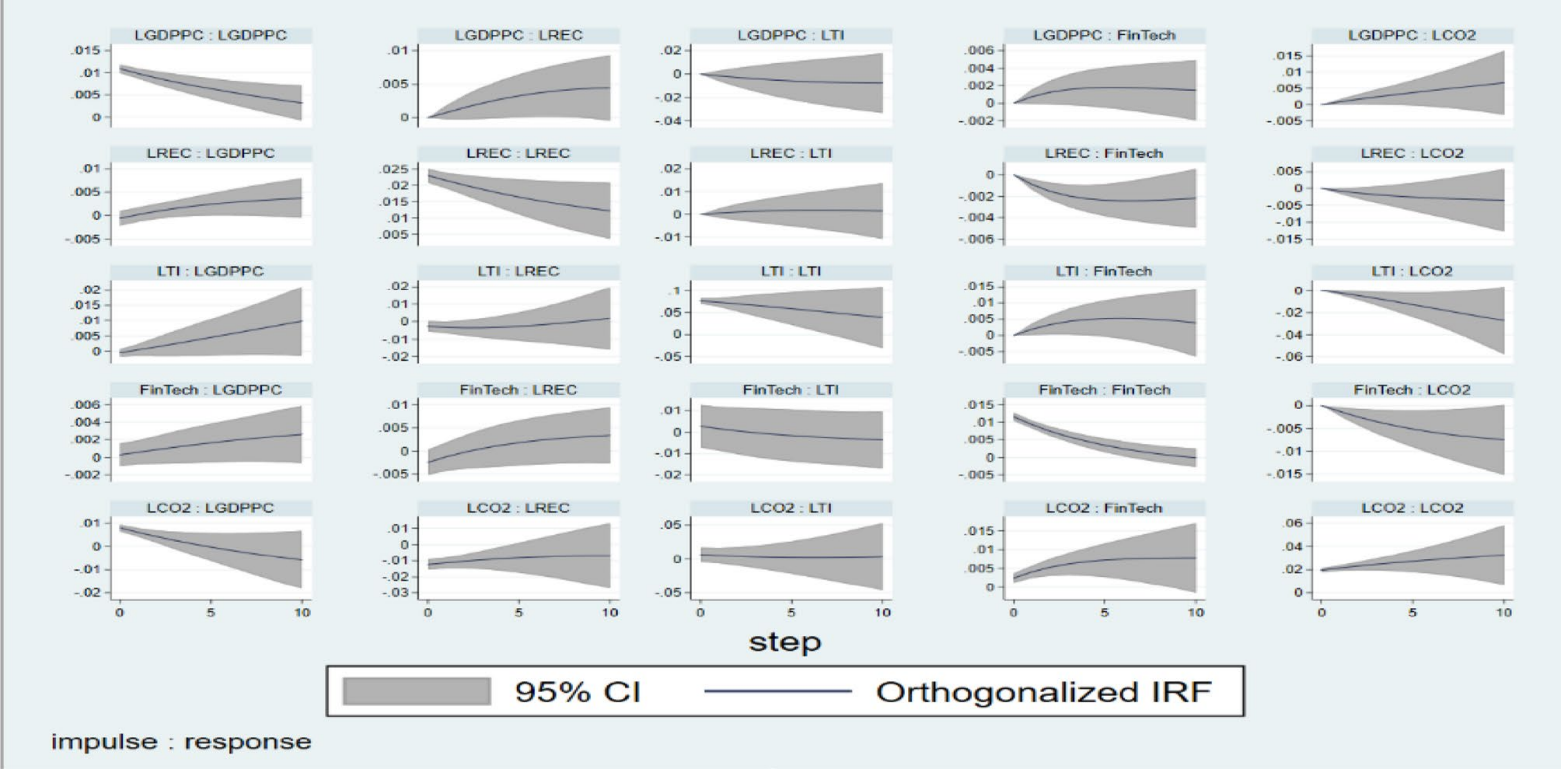
Impulse Response for 10-year horizons generated using VAR.

Figure 5 then displays the impulse response function (IRF) analysis. IRFs highlight the interdependence of all variables, provide insight into how shocks propagate across a system, and investigate if modifications to one variable have a cascade effect on other variables. From the IRFs, it was observed that FinTech has positive impact on GDPPC and REC, which indicates that rising adoption of financial technologies will not only increase the economic growth but also supports the green energy consumption. Previous literature has also observed the positive impact of FinTech on economic growth^24,27,28^. FinTech primarily appears to enhance economic growth by enhancing financial inclusion, increasing productivity, and enabling more seamless financial transactions. For renewable energy consumption, FinTech can support energy technology innovation, crowdsourcing renewable projects, or enabling green energy investments through digital platforms, thereby, increasing the funding and market access for renewable energy sources. FinTech may be a major factor in advancing environmental sustainability and indicating a move toward environmentally friendly technologies^59,60^. Similarly, findings depict decrease in CO_2_ with the rise of FinTech, indicating that environmental sustainability is improved with the technological advancements in FinTech sector. This is consistent with previous studies by^12,39,61,62^. Fintech’s ability to lower emissions is reinforced by the fact that decentralized finance, blockchain, and more digital payments have reduced the need for physical infrastructure. Furthermore, FinTech may encourage green financing, green investments, and environmental greening by assisting businesses and consumers in making eco-friendly choices.

Similar are the findings of technological innovation. With the rise in TI, there is positive impact on GDPPC and REC, however, this impact is negative in case of CO_2_. This indicates that investment in technological innovation exerts positive impact on economic growth, shift to cleaner energy sources, and strengthens the move towards the environmental sustainability. Firms and industry sectors benefit from increased production, improved efficiency, and new opportunities brought about by investments in TI. Technological advancements that expand markets, boost already-existing sectors, and boost overall financial performance may be the source of this growth^44,45^. Previous study suggests that one of the main forces behind the transition to sustainable energy is technological advancements^63,64^. Technological innovation helps with the developments in energy-efficient technology that lower overall energy consumption, or breakthroughs in renewable energy technologies like solar, wind, and energy storage systems, and improves the affordability, effectiveness, and scalability of green technology. Further, by making it possible for more effective industrial methods, cleaner energy consumption, and improved carbon emission control, technological advancement is supporting environmental sustainability^53,54,65^. For instance, developments in low-carbon inventions, clean technology, and energy efficiency help to lower the total carbon footprint of people and businesses.

**Fig. 6:**
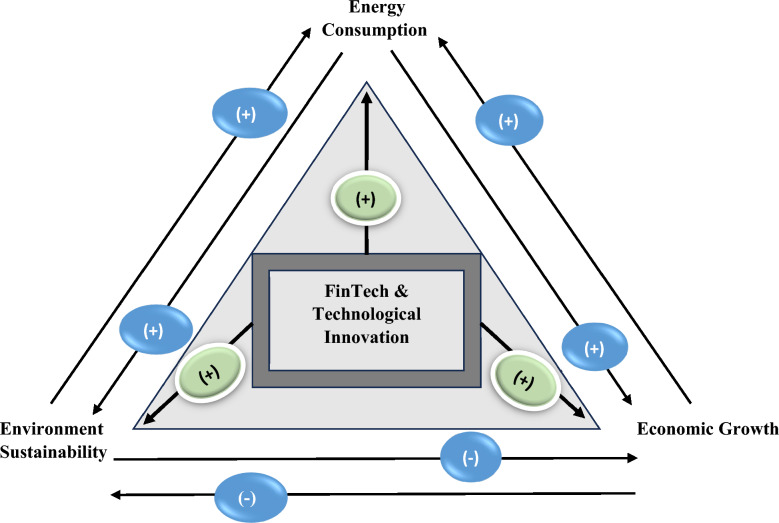
Graphical presentation of result findings.

Further, our results reflect the positive impact of GDPPC on REC depicting that the rising economic growth increases the renewable energy consumption. This can be the result of improved access to green technologies or increased incomes, which allow economies to spend more in sustainable energy to fulfil their expanding energy needs^66,67^. Further, the impact of REC on GDPPC is positive whereas the impact of REC on CO_2_ is negative. These findings show that increase in renewable energy consumption can increase economic growth without increasing carbon emissions. This suggests that consuming renewable energy can help achieve economic growth without sacrificing environmental sustainability^68,69^. This positive effect on renewable energy on environment is also highlighted by^14,19^. To add further, the analysis also sheds light on the impact of CO_2_ on GDPPC and REC. The findings point to the negative impact on economic growth due to rising carbon emissions and more or less stable impact on REC of rising carbon emissions. Growth may eventually be slowed down as emissions increase because the long-term financial costs of climate change and environmental degradation may exceed the short-term gains^40,41^. In addition, the consistent effect of CO_2_ emissions on REC indicates that long-term strategic objectives, technology advancements, and regulatory support ultimately push the adoption of renewable energy. It also illustrates how energy transitions might not be immediately responsive to short-term variations in CO_2_ levels and could take some time. The Graphical presentation of result findings is provided in Fig. 6.

## Conclusion and policy implication

This study investigates the role played by FinTech and technological innovations towards energy, growth and environment nexus for G-20 nations. The period of study ranges from 2005 to 2022 and employs panel VAR approach to examine the interconnection among the variables. From the analysis, it was observed that FinTech and technological innovation have been found to have a positive link with GDPPC, REC, and a decrease in CO_2_ emissions, implying that they play an important role in fostering sustainable development. The findings indicate that technical improvements in FinTech not only promotes economic growth, but also contributes to environmental sustainability by facilitating green energy adoption and lowering carbon footprints. In addition, the findings suggest that while GDPPC encourages REC, it simultaneously increases CO_2_, which points to a critical challenge that when economies grow, the demand for more fossil fuels also increases, potentially leading to larger carbon emissions unless proactive actions are implemented. Further, increasing the use of REC not only helps to reduce CO_2_ emissions but also promotes GDPPC, demonstrating that sustainable development is achievable. However, rising CO_2_ have a negative impact on GDPPC, underscoring the long-term costs of environmental degradation and the need for sustainable economic development.

From a policy standpoint, it is critical to underline the need of strengthening banking institutions’ ability to provide green credit. FinTech platforms can help simplify renewable energy investments by connecting investors with projects and offering risk assessment and due diligence tools. Policy should prioritize creating regulatory sandboxes and foster collaboration between traditional financial institutions and FinTech companies. FinTech can ease the issue and trading of green bonds and other sustainable assets, making it easier for businesses and authorities to raise funds for green initiatives. Further, in the context of G-20, aligning guidelines amongst these nations may render cross-border green investments more feasible and boost the use of renewable energy technologies. Developed G20 nations can leverage each other’s experience in executing green finance initiatives and fostering technological innovation, and they can assist developing nations in transitioning to a low-carbon economy.

### Limitation and future scope of the study

While this study offers valuable insights into the dynamic relationship between FinTech, technological innovation, economic growth, renewable energy consumption, and environmental sustainability within G-20 nations, several limitations should be acknowledged: First, this study focuses exclusively on G-20 nations, which, while significant global actors, may not represent the full spectrum of economic or technological development globally. The findings may not be generalizable to smaller economies or non-G20 developing nations, where FinTech penetration and environmental challenges differ. Second, G-20 countries have diverse economic structures, energy policies, and regulatory environments. This heterogeneity may mask country-specific dynamics that could offer more nuanced insights if analyzed individually. Third, due to limitations in direct measures, certain constructs, such as FinTech development and technological innovation, were represented using proxy indicators. Fourth, global events such as financial crises, pandemics (e.g., COVID-19), or geopolitical tensions during the study period may have influenced energy use, innovation patterns, and economic growth in ways not fully accounted for in the model.

Future research could extend the analysis to include non-G20 developing and emerging economies. This would offer a more comprehensive global perspective. While this study takes a macroeconomic perspective, future studies could adopt a micro-level approach by investigating firm-level or household-level data. This would help understand how FinTech adoption directly influences energy choices, financial behavior, and investment in green technologies. While the panel VAR model provides valuable insights into interrelationships, future studies could explore other dynamic modelling techniques such as panel cointegration, structural VAR (SVAR), or machine learning models to improve predictive capabilities and capture non-linear relationships.

## Data Availability

The datasets used and/or analysed during the current study available from the corresponding author on reasonable request.

## Abbreviations

CO_2_: Carbon emissions
GDPPC: Gross domestic product per capita
REC: Renewable energy consumption
TI: Technological innovation
Panel VAR: Panel vector autoregressive
